# Neovascular age-related macular degeneration in which exudation predominantly occurs as a subretinal fluid during anti-vascular endothelial growth factor treatment

**DOI:** 10.1038/s41598-022-07108-4

**Published:** 2022-02-24

**Authors:** Han Joo Cho, Mi Yeon Song, Wontae Yoon, Jihyun Yoon, Seung Kwan Na, Jihyun Lee, Jaemin Kim, Jong Woo Kim

**Affiliations:** Kim’s Eye Hospital, Konyang University College of Medicine, 156, 4ga, Yeongdeungpo-dong, Yeongdeungpo-gu, Seoul, South Korea

**Keywords:** Macular degeneration, Outcomes research

## Abstract

We investigated the characteristics of neovascular age-related macular degeneration (AMD) in which exudation predominantly occurs as a subretinal fluid (SRF) during anti-vascular endothelial growth factor (VEGF) treatment. A total of 509 treatment-naïve neovascular AMD patients treated with anti-VEGF for 24 months were retrospectively analyzed. The baseline characteristics to determine the odds of occurrence of SRF alone were evaluated using multivariate modeling. SRF was the sole manifestation of lesion activity in 209 (40.9%) eyes during follow-up. The visual outcome of eyes with only SRF occurrence during follow-up was comparable to that of eyes without exudative recurrence. In addition, the incidence of macular atrophy was significantly lower in eyes with only SRF occurrence (9.6%, 20 of 208 eyes) than in eyes without exudative recurrence (16.7%, 9 of 54 eyes, *P* = 0.018). Multivariate analysis revealed that better best-corrected visual acuity (BCVA) at baseline (odds ratio [OR], 0.306; *P* = 0.001), presence of SRF alone at baseline (OR, 5.256; *P* < 0.001), lower pigment epithelial detachment (PED) height (less than 100 µm; OR, 4.113; *P* = 0.025), and aneurysmal type 1 macular neovascularization (MNV) (OR, 2.594; *P* = 0.002) were associated with an increased likelihood of SRF occurrence during follow-up. In conclusion, the eyes with only SRF occurrence during anti-VEGF treatment showed more favorable visual outcomes and a lower incidence of macular atrophy. The baseline characteristics, including better baseline BCVA, presence of SRF alone at baseline, lower PED height, and MNV subtype, might influence the predominant development of SRF during anti-VEGF treatment.

## Introduction

Age-related macular degeneration (AMD) is the leading cause of central vision loss among elderly individuals in developed counties^1^. Neovascular AMD is an advanced form of AMD, characterized by the development of macular neovascularization (MNV) which is subject to leakage and hemorrhage^2^. For over a decade, intravitreal anti-vascular endothelial growth factor (VEGF) injections are the first-line treatment for neovascular AMD^3^.

Optical coherence tomography (OCT) has been used as a critical tool for assessing the activity of MNVs. Fluids from active MNVs can be identified as hyporeflective lesions in multiple retinal compartments, including intraretinal, subretinal, and sub-retinal pigment epithelial (RPE) spaces, on OCT^4^. In general, residual or newly developed fluid on OCT is controlled with additional anti-VEGF injections.

The association between the fluid type on OCT and visual outcomes has been reported by the post hoc analysis of multiple clinical trials including the Comparison of Age-Related Macular Degeneration Treatments Trials (CATT)^5^ and VEGF Trap-Eye: Investigation of Efficacy and Safety in Wet AMD (VIEW 2)^6^. Intraretinal fluid (IRF) is associated with worse baseline visual acuity (VA) and worse visual outcomes after anti-VEGF treatment^5,6^. In contrast, subretinal fluid (SRF) is associated with better baseline VA and better visual outcomes after anti-VEGF treatment. Furthermore, SRF is associated with a lower incidence of macular atrophy (MA). In this context, recent research has suggested that residual SRF can be tolerated and monitored without intensive anti-VEGF treatment^4,7^. The FLUID trial demonstrated that SRF up to 200 µm at the foveal center does not entail worse visual outcomes if left untreated^8^.

Considering the impact of SRF on prognosis, a better visual outcome can be expected if exudative changes include only SRF occurrence during anti-VEGF treatment. To date, there have been no sufficient reports regarding the neovascular AMD phenotype, in which SRF predominantly occurs during long-term anti-VEGF treatment. Moreover, the evaluation of macular fluid in the vast majority of studies has mainly focused on the presence or absence of SRF or IRF, rather than longitudinal analysis. The purpose of the study is to analyze the “SRF-only” phenotype in neovascular AMD patients who underwent anti-VEGF treatment for 24 months and identify the baseline characteristics that correlated with the development of only SRF.

## Materials and methods

Data were collected retrospectively by screening the AMD database of Kim’s Eye Hospital. Patients who were diagnosed with neovascular AMD and treated between March 2016 and February 2019 were recruited. Our study was conducted in accordance with the principles of the Declaration of Helsinki. This study was approved by the Institutional Review Board of Kim’s Eye Hospital, who waived the requirement for informed consent.

### Study population

Subjects who met the following inclusion criteria were enrolled after database screening: (1) over 50 years old; (2) neovascular AMD confirmed with spectral-domain OCT (SD-OCT), fluorescein angiography (FA), and/or indocyanine green angiography (ICGA) at the first visit; (3) no previous treatment before diagnosis; (4) treatment with anti-VEGF (ranibizumab [0.5 mg/0.05 mL] or aflibercept [2 mg/0.05 mL]); and (5) completed follow-up for more than 24 months.

The exclusion criteria were as follows: (1) type 3 MNV (also known as retinal angiomatous proliferation); (2) end-stage conditions presenting disciform scars or fibrosis involving the fovea at the first diagnosis; (3) presence of RPE tear at baseline or development of RPE tear during the follow-up period; and (4) concomitant retinal vascular disorders, such as diabetic retinopathy or retinal vein occlusion.

As a routine practice, all patients received three consecutive monthly intravitreal injections of anti-VEGF. After the initial loading injections (3 injections within 90 days of the first injection), patients were treated with a pro-re-nata (PRN) or treat-and-extend (T&E) regimen. For some patients, the treatment regimen was switched from PRN to T&E or vice versa at the physician's discretion during the study period. Standardized examinations including best-corrected VA (BCVA), fundus examination, and SD-OCT were performed at every visit for all patients. Additional FA/ICGA, OCT angiography, or autofluorescence (AF) imaging was performed at the physician’s discretion during follow-up.

### Data analysis

To identify which neovascular AMD eyes developed only SRF during the 2-year anti-VEGF treatment, the fluid features were evaluated through the analysis of all OCT scans of the enrolled subjects. Four retinal specialists at Kim’s Eye Hospital Reading Center (W.Y., J.Y., S.K.N., and J.L.) reviewed the OCT and FA/ICGA images independently and in a masked manner. SRF was identified on SD-OCT images as a hyporeflective space between the posterior boundary of the neurosensory retina and retinal pigment epithelium (RPE) reflection. IRF was defined as a hyporeflective space within the neurosensory retina on SD-OCT images. The presence of other macular morphologies, including retinal hemorrhage and subretinal hyperreflective material (SHRM)^9^, was also evaluated. Development of only SRF during the maintenance phase was defined as the identification of SRF on SD-OCT without other fluid features, including IRF, SHRM, or retinal hemorrhage.

Pigment epithelial detachment (PED) was defined to maintain the possibility of comparison with clinical trials and our previous investigations. PED was defined as an RPE elevation exceeding 400 µm in width and 75 µm in height or a vertical height of RPE elevation exceeding 200 µm^10–13^. PED showing a focal elevation of the RPE over an optically clear OCT image was defined as serous PED, while PED showing moderately reflective space adherent under the surface of the PEDs on an OCT image was defined as fibrovascular PED^11,14^. When ambiguous cases showed both components of serous and fibrovascular PED on an OCT image, the dominant component of the PED was determined with corresponding FA/ICGA images.

The enrolled neovascular AMD cases were classified as type 1 (sub-RPE choroidal neovascularization [CNV]) MNV and type 2 (sub-retinal CNV) MNV based on multimodal imaging modalities including SD-OCT and FA/ICGA. Cases with a mixture of type 1 and 2 components on SD-OCT images were classified according to the corresponding FA findings; the minimally classic type on FA was considered as type 1 MNV. When characteristic polypoidal lesions were found on ICGA, the type 1 MNV was classified as aneurysmal type 1 MNV/polypoidal choroidal vasculopathy (PCV). Various baseline characteristics, including CNV size, central foveal thickness, and subfoveal choroidal thickness, were manually measured using the built-in software FA/ICGA (Heidelberg Eye Explorer software v. 6.0.9.0; Heidelberg Engineering).

The development of MA was detected on color fundus photography, SD-OCT, infrared reflectance imaging, and AF imaging, similar to the methodology used in our previous studies^15,16^. MA was defined according to the following criteria: (1) hypopigmented area of ≥ 250 µm within the macular vascular arcades, (2) visibility of the underlying choroidal vessels or uniformly reduced signal on AF images, and (3) confirmation of the absence of RPE through OCT images with increased signal transmission in the choroid.

The visual outcome was the change in the BCVA from baseline to 3, 6, 12, 18, and 24 months. The Snellen BCVA was converted to the logarithm of the minimal angle of resolution (logMAR) for statistical analysis. The proportion of patients who gained or lost more than three lines of BCVA compared to the baseline was recorded. The proportion of patients whose BCVA after treatment was ≥ 20/40 or ≤ 20/200 was also recorded. The anatomical outcome was the change in central foveal thickness and incidence of macular atrophy during the study period.

### Statistical analysis

SPSS for Windows, Version 18.0 (SPSS Inc., Chicago, IL, USA) was used for all statistical analyses. Categorical variables were analyzed by the chi-square test. Continuous variables between multiple groups were analyzed by the one-way analysis of variance (ANOVA) with post hoc Bonferroni correction. Stepwise multivariate logistic regression analysis with forward/backward elimination was performed to identify the relationship between the occurrence of SRF alone during the maintenance phase and the patients’ baseline clinical characteristics. A *P*-value of less than 0.05 was considered statistically significant.

## Results

A total of 923 eyes with neovascular AMD treated with anti-VEGF were initially screened from our AMD database during the study period, and 639 eyes had completed 24 months of follow-up. Among them, 130 eyes were excluded for the following reasons: type 3 MNV (61 eyes), RPE tear at baseline or during the study period (31 eyes), retinal vein occlusion or diabetic retinopathy (four eyes), and presenting with end-stage of foveal atrophy or scar at baseline (34 eyes). Ultimately, 509 eyes were included in the analysis.

All patients were South Koreans, and the mean age of the entire study group was 70.1 ± 8.5 years. The mean number of anti-VEGF injections was 9.5 ± 4.1 (range, 3–17) during the 24-month study period. Detailed patient clinical data are presented in Table 1.

**Table 1:** Baseline clinical characteristics of patients with neovascular AMD.

	Total eyes (n = 509)	No exudative recurrence (n = 54)	Presence of only SRF during a 2-year anti-VEGF treatment		*P*
			Yes (n = 208)	No (n = 247)	
**Age (years ± SD)**	70.1 ± 8.5	70.4 ± 9.6	69.7 ± 8.4	70.9 ± 9.2	0.197^a^
**Sex**					0.873^b^
Male, n (%)	275 (54.0%)	28 (51.9%)	115 (55.4%)	132 (53.4%)	
Female, n (%)	234 (46.0%)	26 (48.1%)	93 (44.7%)	115 (46.6%)	
**Mean baseline BCVA (logMAR)** **(Snellen equivalent)**	0.49 ± 0.41 (20/61)	0.52 ± 0.39 (20/66)	0.39 ± 0.32 (20/49)	0.55 ± 0.42 (20/70)	0.021^a^
**Baseline BCVA (logMAR)** **(Snellen equivalent)**					0.143^b^
< 0.40 (20/50)	126 (24.7%)	14 (25.9%)	61 (29.4%)	51 (20.6%)	
0.40 (20/50) to 1.0 (20/200)	267 (52.5%)	30 (55.6%)	107 (51.4%)	130 (52.6%)	
> 1.0 (20/200)	116 (22.8%)	10 (18.5%)	40 (19.2%)	66 (26.7%)	
**Mean central foveal thickness ± SD (µm)**	431 ± 188	448 ± 196	428 ± 201	433 ± 173	0.413^a^
**Mean subfoveal choroidal thickness ± SD (µm)**	264 ± 107	257 ± 121	287 ± 103	252 ± 107	0.097^a^
**Lesion location, n (%)**					0.123^b^
Subfoveal	329 (64.6%)	35 (64.8%)	119 (57.3%)	175 (70.9%)	
Juxtafoveal	119 (23.4%)	10 (18.5%)	60 (28.8%)	49 (19.8%)	
Extrafoveal	61 (12.0%)	9 (16.7%)	29 (13.9%)	23 (9.3%)	
**Mean CNV size ± SD (mm**^**2**^**)**	2.5 ± 2.0	2.2 ± 1.9	2.4 ± 2.2	2.8 ± 1.9	0.173^a^
**Presence of choroidal vascular hyperpermeability, n (%)**^c^	195 (38.3%)	18 (33.3%)	88 (42.3%)	89 (36.0%)	0.284^b^
**Baseline fluid feature, n (%)**					< 0.001^b^
SRF alone	196 (38.5%)	24 (44.4%)	120 (57.7%)	52 (21.1%)	
SRF with other exudation (IRF, hemorrhage^d^, or SHRM)	165 (32.4%)	15 (27.8%)	43 (20.7%)	107 (43.3%)	
Absence of SRF	148 (29.1%)	15 (27.8%)	45 (21.6%)	88 (35.6%)	
**Presence of PED at baseline, n (%)**					
Fibrovascular PED	302 (59.3%)	30 (55.6%)	121 (58.2%)	151 (61.1%)	0.113^b^
Serous PED	112 (22.0%)	9 (16.7%)	50 (24.0%)	53 (21.5%)	0.487^b^
**PED height at baseline (µm)**	281 ± 183	272 ± 233	238 ± 179	288 ± 211	0.002^a^
**MNV subtype**					< 0.001^b^
Type 1	259 (50.9%)	26 (48.1%)	93 (44.7%)	140 (56.7%)	
Aneurysmal type 1/PCV	188 (37.0%)	23 (42.6%)	108 (51.9%)	57 (23.1%)	
Type 2	62 (12.1%)	5 (9.3%)	7 (3.4%)	50 (20.2%)	
**Anti-VEGF agent, n (%)**					0.336^b^
Ranibizumab	121 (23.8%)	13 (24.1%)	55 (26.4%)	53 (21.5%)	
Aflibercept	286 (56.2%)	28 (51.9%)	120 (57.7%)	138 (55.9%)	
Both^e^	102 (20.0%)	13 (24.0%)	33 (15.9%)	56 (22.6%)	
**Treatment regimen, n (%)**					0.136^b^
PRN	249 (48.9%)	35 (64.8%)	99 (47.6%)	115 (46.6%)	
T&E	172 (33.8%)	14 (25.9%)	69 (33.2%)	89 (36.0%)	
Both^f^	88 (17.3%)	5 (9.3%)	40 (19.2%)	43 (17.4%)	
**Mean number of anti-VEGF injections ± SD**	9.5 ± 4.1	8.7 ± 4.7	9.6 ± 5.1	9.4 ± 4.4	0.070^a^

AMD, age-related macular degeneration; BCVA, best-corrected visual acuity; CNV, choroidal neovascularization; IRF, intraretinal fluid; logMAR, logarithm of the minimum angle of resolution; MNV, macular neovascularization; PCV, polypoidal choroidal vasculopathy; PED, pigment epithelial detachment; PRN, pro-re-nata; SD, standard deviation; SHRM, subretinal hyperreflective material; SRF, subretinal fluid; T&E, treat-and-extend; VEGF, vascular endothelial growth factor.

^a^*p*-value by analysis of variance.

^b^*p*-value by chi-square test.

^c^Indocyanine green angiography image was not available for 38 eyes among the total eyes.

^d^Refers to any hemorrhage, not necessarily limited to the lesion, including subretinal and sub-RPE hemorrhage.

^e^Patients switched from an anti-VEGF drug to the other one during the study period; 74 eyes (72.5%) were switched from ranibizumab to aflibercept, while the others were switched from ranibizumab to aflibercept.

^f^Treatment regimen was switched from PRN to T&E in 52 (59.1%) eyes and from T&E to PRN in others (40.9%) during the study period.

### Neovascular AMD in which exudation occurred as SRF alone during the maintenance phase

After the loading injections, macular fluid, including SRF, IRF, SHRM, or hemorrhage, was identified on OCT more than once for most eyes (90.4%, 455 of 509 eyes) during the 24-month anti-VEGF treatment. The remaining eyes (10.6%, 54 of 509 eyes) showed no exudative recurrence after loading injections during the maintenance period. Among the cases showing macular fluid during the study period, 208 (40.9%) eyes showed only SRF as the sole manifestation of lesion activity during the maintenance period.

Several different baseline characteristics were found between the group with exudation manifesting only as SRF during the maintenance period, the group with other exudation, and the group without exudative recurrence. Eyes with only SRF occurrence during follow-up showed significantly better BCVA (0.39 ± 0.32) at baseline than eyes without exudative recurrence and eyes with other exudation (0.52 ± 0.39 and 0.55 ± 0.42, respectively; *P* = 0.021, Table 1). The baseline fluid features were different between the groups; the presence of SRF alone at baseline was more frequent (57.7%) in the group with only SRF during follow-up than in the group without exudative recurrence and the group with other exudation during follow-up (44.4% and 21.1%, respectively; *P* < 0.001, Table 1). The mean PED height (238 ± 179 µm) at baseline was significantly lower for the group with only SRF than for the group without exudative recurrence and the group with other exudation during follow-up (272 ± 233 µm and 288 ± 211 µm, respectively; *P* = 0.002, Table 1). The proportion of MNV subtypes was also different between the groups; aneurysmal type 1 MNV/PCV was more frequent and types 1 and 2 MNV were less frequent in the group with only SRF during follow-up than in the other groups (*P* < 0.001, Table 1).

No significant differences were found between the groups in terms of other baseline characteristics including age, sex, central foveal thickness, subfoveal choroidal thickness, lesion location, CNV size, presence of choroidal vascular hyperpermeability, type of anti-VEGF agent, and injection number (Table 1).

### Visual outcome

**Figure 1:**
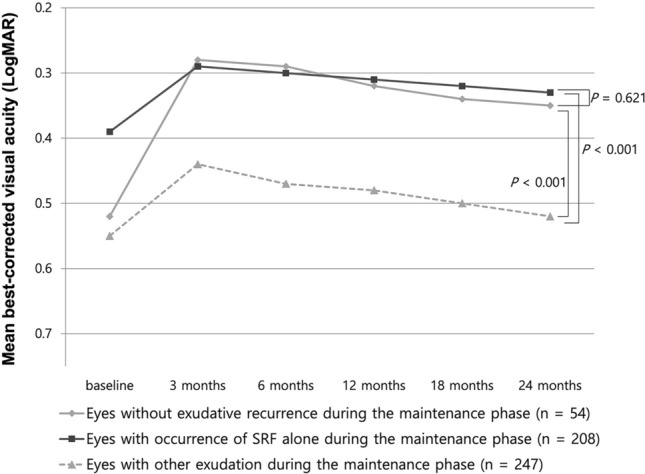
Changes in the mean best-corrected visual acuity (BCVA) expressed as the logarithm of the minimal angle of resolution during the 24-month follow-up, where anti-vascular endothelial growth factor treatment for neovascular age-related macular degeneration (AMD) was employed. At 24 months, the BCVA of eyes with only subretinal fluid (SRF) during the maintenance phase was comparable to that of eyes without exudative recurrence (*P* = 0.621) and significantly better than that of eyes with exudation except for cases of SRF alone during the maintenance phase (Bonferroni correction after one-way analysis of variance, *P* < 0.001).

Visual outcome was correlated with the features of exudation during the 2-year anti-VEGF treatment, as documented by OCT analysis. The time courses of the BCVA changes were analyzed between the no exudate group, the SRF only group, and the other exudation group during follow-up (Fig. 1). The baseline BCVA (0.39 ± 0.32) of eyes with only SRF during the maintenance phase was significantly better than that of the no exudative recurrence group and the other exudation group during follow-up (0.52 ± 0.39 and 0.55 ± 0.42, respectively; *P* = 0.002). After the 24-month anti-VEGF treatment, the visual outcomes were significantly different among the groups (*P* = 0.021, Fig. 1). The BCVA at 24 months of the group with only SRF (0.33 ± 0.29) and the group without exudative recurrence (0.35 ± 0.27) were significantly better than that of the group with other exudation (0.52 ± 0.35; *P* < 0.001, Bonferroni correction, Fig. 1). However, the BCVA (0.33 ± 0.29) of eyes with only SRF during follow-up showed no significant difference compared to that of eyes without exudative recurrence (0.35 ± 0.31; Bonferroni correction, *P* = 0.621).

**Figure 2:**
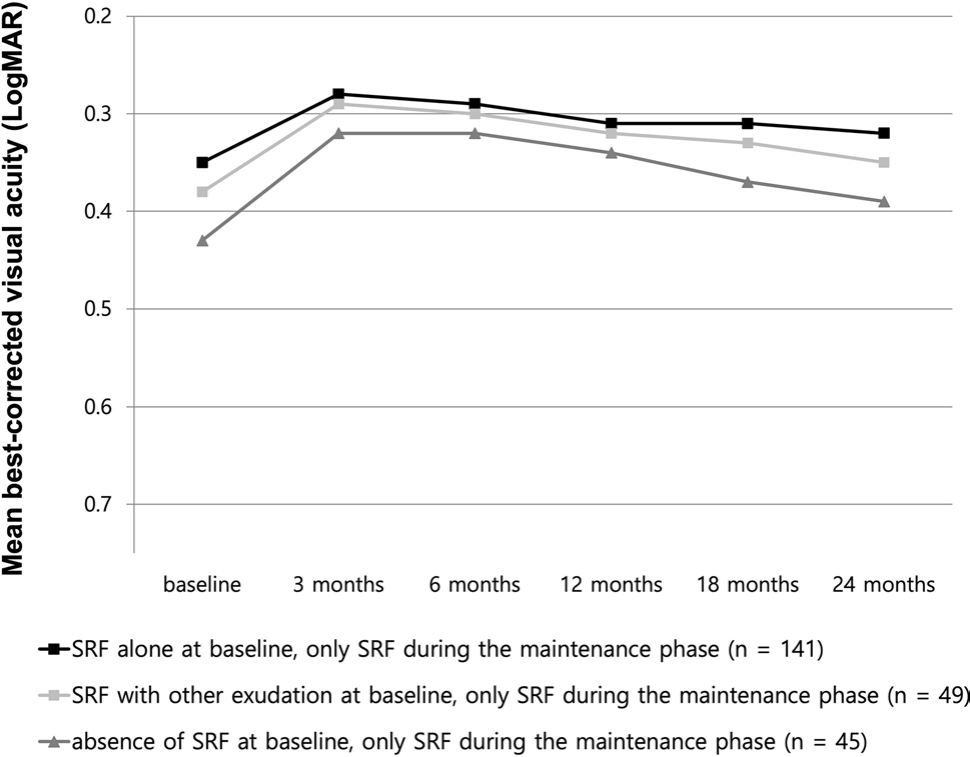
Subgroup analysis for best-corrected visual acuity (BCVA) changes for eyes with only subretinal fluid (SRF) during the maintenance phase. There was no significant difference in BCVA at 24 months irrespective of the baseline fluid characteristics. Even if there was no SRF at baseline, the BCVA of eyes with only SRF during the maintenance phase (gray-dotted line) showed no statistical difference at 24 months compared to that of eyes with only SRF from baseline to 24 months (black solid line) (one-way analysis of variance, *P* = 0.081).

Subgroup analysis for eyes with only SRF during the maintenance phase (208 eyes) showed no significant difference in BCVA at 24 months, irrespective of the baseline fluid characteristics (Fig. 2). Eyes with only SRF at baseline tended to show better BCVA throughout the 24-month anti-VEGF treatment; however, no statistical difference was found among the groups at 24 months; the BCVA (0.38 ± 0.33) of eyes without SRF at baseline showed non-inferiority to that of eyes with SRF alone at baseline and eyes with other fluid features (0.32 ± 0.30 and 0.35 ± 0.29, respectively; *P* = 0.081, Fig. 2).

The proportion of improved VA (≥ 3-line gain of BCVA) at 24 months was not significantly different between eyes without exudative recurrence (33.3%, 18 of 54 eyes), eyes with only SRF (20.2%, 42 of 208 eyes), and eyes with exudation except for cases of SRF alone during follow-up (19.8%, 49 of 247 eyes; *P* = 0.069, Table 2). However, the proportion of worsened VA (≥ 3-line loss of BCVA) at 24 months was significantly lower for eyes with only SRF during follow-up (9.7%, 18 of 208 eyes) than in eyes without exudative recurrence and eyes with exudation except for cases of SRF alone during follow-up (13.0% and 19.4%, respectively; *P* = 0.004, Table 2). The proportion of BCVA ≥ 20/40 or ≤ 20/200 after anti-VEGF treatment was not significantly different among the groups (Table 2).

**Table 2:** Comparisons of treatment outcome according to exudation features during the 2-year anti-VEGF treatment.

	No exudative recurrence group (54 eyes)	Exudative recurrence group with SRF alone (208 eyes)	Other exudative recurrence group (247 eyes)	*P*
**Mean BCVA at 24 months (logMAR [Snellen equivalent])**	0.35 ± 0.27 (20/44)	0.33 ± 0.29 (20/42)	0.52 ± 0.35 (20/66)	0.008^a^
**Mean central foveal thickness at 24 months (µm)**	266 ± 120	292 ± 131	280 ± 124	0.615^a^
**BCVA ≥ 20/40, n (%)**	19 (35.2%)	72 (34.6%)	71 (28.7%)	0.347^b^
**BCVA ≤ 20/200, n (%)**	6 (11.1%)	28 (13.5%)	42 (17.0%)	0.405^b^
**BCVA changes, n (%)**				
Improved ≥ 3 lines (logMAR 0.3)	18 (33.3%)	42 (20.2%)	49 (19.8%)	0.069^b^
Worsened ≥ 3 lines (logMAR 0.3)	7 (13.0%)	18 (9.7%)	49 (19.4%)	0.004^b^
**Development of macular atrophy, n (%)**	9 (16.7%)	20 (9.6%)	47 (19.0%)	0.018^b^

^a^Based on one-way analysis of variance.

^b^Based on chi-square test.

BCVA, best-corrected visual acuity; logMAR, logarithm of the minimum angle of resolution; SRF, subretinal fluid; VEGF, vascular endothelial growth factor.

### Anatomical outcome

**Figure 3:**
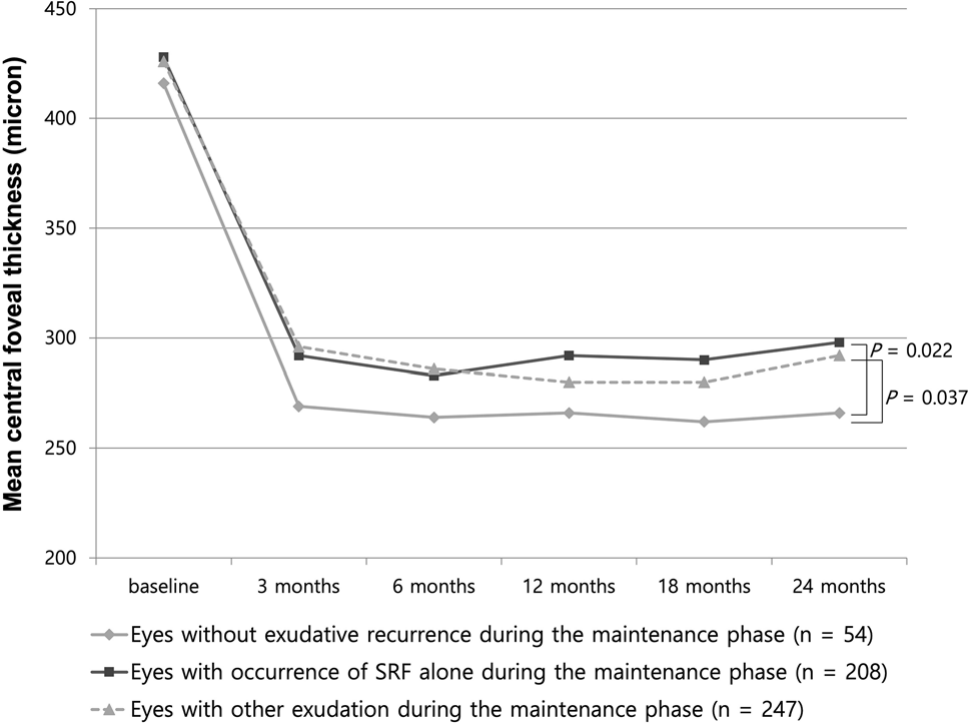
Changes in mean central foveal thickness during 24-month anti-vascular endothelial growth factor treatment for neovascular age-related macular degeneration. The mean central foveal thickness showed a significant decrease from baseline to 24 months in all groups. After the 24-month treatment, central foveal thickness of eyes without exudative recurrence was significantly thinner than that of eyes with occurrence of SRF alone (P = 0.022) and eyes with exudation except for cases of SRF alone (*P* = 0.037; Bonferroni correction after one-way analysis of variance).

The mean central foveal thickness showed a significant decrease during the 24-month follow-up in all groups (Fig. 3). After 24 months of treatment, the central foveal thickness was significantly thinner in eyes without exudation during follow-up (266 ± 177 µm) than in eyes with SRF alone and eyes with exudation except for cases of SRF alone (298 ± 192 µm and 292 ± 188 µm; *P* = 0.022 and *P* = 0.037, respectively; Bonferroni correction after ANOVA, Fig. 3).

The incidence of MA development during the study period differed between the groups. The incidence was significantly lower in eyes with only SRF during follow-up (9.6%, 20 of 208 eyes) than in eyes without exudative recurrence (16.7%, 9 of 54 eyes) and eyes with exudation except for cases of SRF alone during follow-up (19.0%, 47 of 247 eyes; *P* = 0.018, Table 2).

### Odds for the occurrence of only SRF during the maintenance phase

Several baseline characteristics were significantly associated with the predominant occurrence of SRF through multivariate logistic regression analysis (Table 3). Better BCVA at baseline (odds ratio [OR] 0.306; 95% confidence interval [CI] 0.148–0.633; *P* = 0.001) showed an increased odds of occurrence of SRF alone during the maintenance phase. Among the baseline fluid features, the presence of SRF alone (OR 5.256; 95% CI 2.630–8.504; *P* < 0.001) was associated with an increased odds of SRF occurrence during follow-up. However, when other fluid features were present, the odds did not increase despite the presence of SRF at baseline. A lower PED height at baseline was associated with a higher likelihood of SRF occurrence. Specifically, when the PED height was < 100 µm (OR 4.113; 95% CI 2.229–6.662, *P* = 0.025) or $\ge$ 100 µm and < 200 µm (OR 2.002; 95% CI 1.088–4.049, *P* = 0.043), the odds was significantly increased. However, no significant difference was found for PED height $\ge$ 200 µm. The MNV subtype correlated with the odds of SRF occurrence (*P* < 0.001). Aneurysmal type 1 MNV/PCV (OR 2.594; 95% CI 1.428–4.373, *P* = 0.002) was associated with a higher odds and type 2 MNV (OR 0.181; 95% CI 0.050–0.662, *P* = 0.010) was associated with a lower odds than type 1 MNV (Table 3).

**Table 3:** Association between baseline ocular characteristics and occurrence of SRF alone during the 2-year anti-VEGF treatment: logistic regression analysis.

Variable	Univariate analysis		Multivariate analysis		
	OR (95% CI)	*P*	OR (95% CI)		*P*
**Age**	0.841 (0.801–0.972)	0.023	0.982 (0.949–1.016)		0.289
**Sex**^a^	0.972 (0.854–1.077)	0.421			
**Baseline BCVA (logMAR)**	0.232 (0.143–0.379)	< 0.001	0.306 (0.148–0.633)		0.001
**Baseline central foveal thickness**	0.966 (0.822–1.258)	0.464			
**Baseline subfoveal choroidal thickness**	1.011 (1.002–1.023)	0.012	1.008 (0.982–1.033)		0.346
**Lesion location**^a^	0.887 (0.811–1.386)	0.588			
**CNV size**	0.934 (0.830–1.060)	0.235			
**Presence of choroidal vascular hyperpermeability**^a^	1.775 (0.916–2.228)	0.231			
**Baseline fluid feature**^**a**^		< 0.001			< 0.001
SRF alone	6.087 (3.777–9.809)	< 0.001	5.256 (2.630–8.504)		< 0.001
SRF with IRF, retinal hemorrhage, and/or SHRM	1.008 (0.614–1.653)	0.871	1.087 (0.755–1.404)		0.702
Absence of SRF	1.00		1.00		
**Presence of PED**^**a**^		0.004			0.168
None	1.00		1.00		
Fibrovascular PED	0.512 (0.343–1.264)	0.092	0.771 (0.558–1.235)		0.111
Serous PED	1.144 (1.006–1.488)	0.008	1.482 (0.980–2.111)		0.075
**PED height at baseline (µm)**^**a**^		0.001			0.031
< 100	3.825 (2.060–7.102)	< 0.001	4.113 (2.229–6.662)		0.025
100–199	2.236 (1.066–4.013)	0.027	2.002 (1.088–4.049)		0.043
200–299	1.863 (1.039–3.340)	0.120	1.263 (0.945–2.664)		0.216
300–399	1.811 (0.829–2.674)	0.145	1.811 (0.899–2.674)		0.229
≥ 400	1.00		1.00		
**MNV subtype**^**a**^		< 0.001			< 0.001
Type 1	1.00		1.00		
Aneurysmal type 1/PCV	2.248 (1.525–3.313)	< 0.001	2.594 (1.428–4.373)		0.002
Type 2	0.199 (0.091–0.435)	< 0.001	0.181 (0.050–0.662)		0.010
**Treatment regimen**	0.882 (0.715–1.337)	0.315			
**Anti-VEGF agent (ranibizumab or aflibercept)**^a^	0.977 (0.872–1.113)	0.569			
**Number of injections**	1.017 (0.980–1.123)	0.345			

AMD, age-related macular degeneration; BCVA, best-corrected visual acuity; CI, confidence interval; CNV, choroidal neovascularization; logMAR, logarithm of the minimum angle of resolution; MNV, macular neovascularization; OR, odds ratio; PCV, polypoidal choroidal vasculopathy; PED, pigment epithelial detachment; SRF, subretinal fluid; VEGF, vascular endothelial growth factor.

The R-squared of the model was 0.563.

^a^Categorical variable.

## Discussion

Fluid accumulation secondary to the disruption of the blood–retina barrier is frequently caused by the neovascular activity of MNV. The breakdown of the outer blood–retinal barrier leads to exudation into the subretinal space (SRF), and breakdown of the external limiting membrane leads to exudation into the neurosensory retina (IRF)^17^. Infrequently, VEGF overexpression may induce IRF independently in case of type 3 MNV^18^. IRF leads to alteration of bipolar axons, which cause neurosensory damage^19^. Importantly, the neurosensory damage caused by IRF could not be reversed by adding an intensive anti-VEGF treatment^5^. Thus, according to many researches, IRF should not be tolerated and should be treated more aggressively than SRF^20,21^. On the contrary, SRF is not detrimental to visual outcome, to a certain extent, and residual SRF is associated with a lower incidence of macular atrophy^10,22^.

The rationale for the positive effect of SRF on visual function is unclear. One proposed hypothesis is that the presence of SRF can act as a trophic support to the outer retina, which promotes RPE and outer retinal survival^21^. This hypothesis is proposed from the perspective that type 1 CNV is a compensatory response to local ischemia, and therefore, the derived SRF may contain oxygen and nutrients^4,21^. Furthermore, the presence of SRF has been associated with a lower incidence of macular atrophy, which could explain in part the reason for good VA in eyes with SRF^23^. Therefore, considering the impact of SRF on prognosis, better visual results can be predicted if exudative changes include only SRF occurrence during anti-VEGF treatment.

In the current study, 40.9% of the total eyes manifested with SRF alone as the disease activity after the loading phase during a 2-year anti-VEGF treatment. The visual outcome of the eyes with SRF alone during follow-up was comparable to that of the eyes without exudative recurrence and even tended to be better. On the other hand, the visual gain of the SRF only group was relatively lower than that of the group without recurrence; this might have resulted from the ceiling effect, considering that the baseline VA was significantly better for the SRF-alone group.

There were several visual and anatomical outcomes associated with better prognosis in the SRF-only group than in the other groups. Specifically, the proportion of patients with worsened VA of more than three lines was lower in the group with SRF alone during follow-up than that in the group without exudative recurrence for 24 months. In addition, the incidence of macular atrophy in the group with only SRF was significantly lower than that in the group without exudative recurrence. Furthermore, even in the absence of SRF at baseline, when SRF predominantly occurred during the maintenance phase, the visual outcome was non-inferior to that of the eyes with SRF at baseline. Thus, our results suggest that the prediction of which type of fluid would be predominantly developed in neovascular AMD during follow-up could be helpful in predicting prognosis after anti-VEGF treatment.

Several baseline characteristics were identified as factors associated with the predominant occurrence of SRF during anti-VEGF treatment in our study. The baseline fluid characteristics showed the highest correlation with the occurrence of SRF alone during follow-up. The presence of SRF alone at baseline was associated with an approximately 5.2-fold higher likelihood of predominant occurrence of SRF during follow-up. The presence of other fluid features, including IRF, hemorrhage, or SHRM, was not associated with the predominant occurrence of SRF during the maintenance phase, irrespective of the presence of SRF at baseline.

A lower PED height of < 200 µm was associated with an increased odds of SRF occurrence in our study. Specifically, for cases with shallow PED of < 100 µm, the likelihood of the predominant occurrence of SRF during follow-up increased by approximately four times. Recent studies have reported that fibrovascular PED is associated with an increased risk of IRF development compared to serous PED^10,24^. However, no significant association was found between the predominant occurrence of SRF and PED subtype in our study. Our results suggest that the features of PED at baseline can be helpful in predicting the occurrence of SRF during anti-VEGF treatment.

The MNV subtype was identified as another factor associated with the occurrence of SRF alone during follow-up in our study. Compared to type 1 MNVs, aneurysmal type 1 MNV/PCV was more likely and type 2 MNV was less likely to be associated with the predominant occurrence of SRF. It has been reported that SRF is more frequent and IRF is less common in patients with aneurysmal type 1 MNV/PCV at baseline than in those with typical neovascular AMD^10^. Our results identified that the occurrence of SRF is more frequent in patients with aneurysmal type 1 MNV/PCV not only at baseline but also during anti-VEGF treatment than those with other types of MNV. Approximately half (49.5%, 103 of 188 eyes) of the enrolled eyes with aneurysmal type 1 MNV/PCV showed exudation as SRF alone during the maintenance phase.

Type 3 MNV is a subtype of neovascular AMD; however, such cases were not included in our study for several reasons. The current concept of the development of type 3 MNV is that it originates from the deep capillary plexus rather than the choroid and descends downward toward the RPE as it progresses^15^. Hence, IRF is present in nearly every case of type 3 MNV^25,26^. Given that IRF is a poor prognostic factor for neovascular AMD, the impact of macular fluid on visual outcome in type 3 MNV could be somewhat different from that in other types of MNV. A recent investigation regarding macular fluid in type 3 MNV reported that fluid-based visual prognostication was different from that in other forms of MNV and that IRF was associated with good visual outcomes^26^.

Our study has several limitations. First, due to its retrospective nature, the treatment regimen and anti-VEGF therapy were selected at the physician’s discretion. If the study was conducted with a fixed anti-VEGF injection schedule in clinical trials, the results could be different. However, previous investigations have shown that more than 90% of retinal specialists use a personalized treatment regimen based on PRN or T&E for the management of neovascular AMD^27^. We expect our data to reflect real-world practice, which could be helpful for physicians. Second, the proportion of eyes with only SRF during the study follow-up could also be different from that in clinical trials because of the relatively higher proportion of patients with aneurysmal type 1 MNV/PCV enrolled in our study. However, this issue could not be identified because most clinical trials, including the VIEW^13^, CATT^28^, and EXCITE studies^29^, have mainly focused on the presence of a specific fluid at a specific time point. Third, we did not evaluate SRF quantitatively. A recent post hoc analysis of the FLUID study has indicated that the volume of IRF and SRF could be quantified with an artificial intelligence algorithm^30^. The exact association between the VA changes and qualitative analysis of SRF needs to be investigated in the future.

In conclusion, the visual outcome of neovascular AMD patients who showed only SRF as the sole manifestation of lesion activity during the 24-month anti-VEGF treatment was comparable to that of patients without exudative recurrence. The baseline characteristics, including better baseline BCVA, presence of SRF alone at baseline, lower PED height, and MNV subtype, might influence the predominant development of SRF during anti-VEGF treatment. Identifying these baseline predictive factors for the predominant occurrence of SRF during anti-VEGF treatment might be clinically relevant as this information allows clinicians to better categorize patients and provide more tailored anti-VEGF treatments for patients.
